# Explorations on the antiviral potential of zinc and magnesium salts against chikungunya virus: implications for therapeutics

**DOI:** 10.3389/fcimb.2024.1335189

**Published:** 2024-06-04

**Authors:** Kusuma Sai Davuluri, Shridhar Shukla, Mahadeo Kakade, Sarah Cherian, Kalichamy Alagarasu, Deepti Parashar

**Affiliations:** ^1^ Dengue and Chikungunya Group, ICMR-National Institute of Virology, Pune, India; ^2^ Bioinformatics Group, ICMR-National Institute of Virology, Pune, India; ^3^ Academy of Scientific and Innovative Research (AcSIR), Ghaziabad, India

**Keywords:** zinc, magnesium, chikungunya virus, antiviral agents, therapeutics

## Abstract

**Background:**

Chikungunya virus (CHIKV), which causes chikungunya fever, is an arbovirus of public health concern with no approved antiviral therapies. A significant proportion of patients develop chronic arthritis after an infection. Zinc and magnesium salts help the immune system respond effectively against viral infections. This study explored the antiviral potential of zinc sulphate, zinc acetate, and magnesium sulphate against CHIKV infection.

**Methods:**

The highest non-toxic concentration of the salts (100 µM) was used to assess the prophylactic, virucidal, and therapeutic anti-CHIKV activities. Dose-dependent antiviral effects were investigated to find out the 50% inhibitory concentration of the salts. Entry bypass assay was conducted to find out whether the salts affect virus entry or post entry stages. Virus output in all these experiments was estimated using a focus-forming unit assay, real-time RT-PCR, and immunofluorescence assay.

**Results:**

Different time- and temperature-dependent assays revealed the therapeutic antiviral activity of zinc and magnesium salts against CHIKV. A minimum exposure of 4 hours and treatment initiation within 1 to 2 hours of infection are required for inhibition of CHIKV. Entry assays revealed that zinc salt affected virus-entry. Entry bypass assays suggested that both salts affected post-entry stages of CHIKV. In infected C57BL6 mice orally fed with zinc and magnesium salts, a reduction in viral RNA copy number was observed.

**Conclusion:**

The study results suggest zinc salts exert anti-CHIKV activity at entry and post entry stages of the virus life cycle, while magnesium salt affect CHIKV at post entry stages. Overall, the study highlights the significant antiviral potential of zinc sulphate, zinc acetate, and magnesium sulphate against CHIKV, which can be exploited in designing potential therapeutic strategies for early treatment of chikungunya patients, thereby reducing the virus-associated persistent arthritis.

## 1 Introduction

Chikungunya virus (CHIKV), an arthritogenic virus belonging to the family Togaviridae and genus Alpha virus, causes chikungunya fever (CHIKF). Infected patients recover within a week; however, chronic arthritis in some might persist for weeks to a few months. Currently, there are no antivirals approved for treating CHIKF, and the treatment focuses on supportive care. Supportive care measures include the administration of pain relievers (like acetaminophen), anti-inflammatory drugs (avoiding nonsteroidal drugs due to the potential risk of bleeding), and maintaining adequate hydration (da Cunha and Trinta, 2017). Viral persistence has been shown to be associated with arthritis (Zhang et al., 2022), and reducing the viral load might contribute to preventing the development of chronic arthritis. Currently, several plant-based extracts as well as FDA-approved drugs are being explored for their efficacy in suppressing CHIKV infections (Patil et al., 2021; Alagarasu et al., 2022; Kasabe et al., 2023). Though the recent approval of Ixchiq, the first chikungunya vaccine, by the U.S. Food and Drug Administration represents a significant advancement in preventive measures (https://www.fda.gov/, assessed on December 9, 2023), it is imperative to acknowledge that the development of new medicines remains crucial. Despite the availability of vaccines, the ongoing need for effective antiviral treatments and therapeutics underscores the importance of a multifaceted approach to combating viral infections.

Studies have reported that zinc salts, an important micronutrient, exert *in-vitro* antiviral activity against different viruses. Zinc interferes with human papillomavirus replication by inhibiting the synthesis of crucial viral proteins (Read et al., 2019). Zinc chloride inhibits the production of infectious virus particles in chicken embryo fibroblast cells infected with Sindbis virus (Bracha and Schlesinger, 1976). Zinc ions also inhibit Semiliki Forest virus (SFV) liposome fusion and particularly inhibit the formation of the post fusion E1 trimer in wild-type SFV (Corver et al., 1997; Liu and Kielian, 2012). Studies have examined the role of zinc supplements in reducing the severity and duration of viral infections, especially for the common cold and certain coronaviruses (Marreiro et al., 2022). Oral zinc supplementation has been reported to enhance the recovery rate and reduce the incidence of severity as an adjunctive treatment for rotavirus enteritis in infants (Telmesani, 2010; Jiang et al., 2016). Chelation of zinc abrogates dengue virus replication (Kar et al., 2019). Zinc ions can penetrate cell membranes and accumulate within infected cells. Within the cell, zinc exerts its antiviral effects by disrupting viral replication mechanisms and impeding the formation and release of new virus particles (Xia et al., 2021). Holzmann et al. (1988) explored the clinical efficacy of combinations of heparin and ZnSO_4_ against the herpes simplex virus (HSV). Despite its clinical effectiveness, the underlying mechanism of the alleged antiherpetic activity remained unclear. Studies investigating the inhibitory effects of zinc salts on HSV replication indicated that zinc ions inhibited HSV DNA polymerase at a lower concentration compared to cellular DNA polymerases α and β in cell homogenates (Fridlender et al., 1978). Additionally, zinc also acts as an antioxidant, protecting cells from oxidative damage and maintaining cellular integrity and function, potentially reducing the impact of viruses on host cells (Cuajungco et al., 2021).

Magnesium is an essential mineral with potential antiviral properties. Evidence suggests that magnesium can modulate immune responses and influence viral replication, although it is not typically considered a direct antiviral agent (Mizutani et al., 1994). By aiding immune cell activation, cytokine production, and antibody response, magnesium supports a well-balanced immune system crucial for effective defense against viral infections (Tam et al., 2003).

The potential impact of supplementing zinc and magnesium, both *in vitro* and *in vivo*, on CHIKV has not been studied. In the present study, the antiviral potential of zinc sulphate, zinc acetate, and magnesium sulphate against CHIKV replication was explored using a range of experimental approaches.

## 2 Materials and methods

### 2.1 Ethics statement

Animal experiments were performed within a biosafety level-2 animal facility at the ICMR-National Institute of Virology, Pune. The research protocol was approved by both the Institutional Animal Ethics Committee (IAEC) and the Institutional Biosafety Committee. The housing and care of animals adhered strictly to the guidelines established by the Committee for Control and Supervision of Experiments on Animals (CCSEA) (IAEC number CHK 1501, approved on December 20, 2017).

### 2.2 Virus and cell line used

The CHIKV strain (061573, Andhra Pradesh, India), was used in all the experiments. Vero CCL-81 cells (ATCC^®^ CCL81™) were grown in minimal essential medium (MEM) (Himedia^®^, India) containing 10% fetal bovine serum (FBS) (Gibco™, USA) and antimycotic antibiotic solution (Sigma Aldrich^®^, USA) at 37°C with 5% CO_2_. Zinc sulphate (Product No. Z4750, purity ≥99.0% based on titration with EDTA), zinc acetate (Product No. 379786, purity ≥99.999% based on trace metal analysis), and magnesium sulphate (Product No. M2643, purity > 98% based on titration with EDTA) were procured from Sigma-Aldrich Chemicals for use in the study. The compounds were dissolved in double-distilled water and diluted in the culture medium before each assay.

### 2.3 Cell viability assessment

The cytotoxic effects of the zinc and magnesium salts were assessed as described previously (Patil et al., 2021). Briefly, Vero CCL-81 cells (3.5×10^4^ cells per well) cultured in 96-well micro-titre plates were treated with a range of concentrations (450 μM to 2.5 μM) of zinc and magnesium salts. Cell viability was investigated using the 3-(4,5-dimethythiazol-2-yl)-2,5-diphenyll tertrazolium bromide (MTT) reagent (Sigma-Aldrich™, USA) after 24, and 48 hours. CC50, the concentration of the compound needed to reduce the viability of cells by 50%, was computed using non-linear regression analysis.

### 2.4 Screening of anti-CHIKV activity of zinc and magnesium salts

To assess the antiviral potential of zinc and magnesium salts, the highest concentrations of the salts that did not exhibit toxicity were chosen. This assessment was conducted under three distinct scenarios: prior to infection (pre-treatment), during infection (co-treatment), and after infection (post-treatment). In each case, 2×10^5^ cells *per* well were seeded in a 24-well plate and infected with a multiplicity of infection (MOI) of 0.01. After infection, free virus particles were removed by washing twice with 1x sterile phosphate-buffered saline (PBS), and the plates were then incubated for 24 hours in presence of salts or only MEM with FBS.

In the pre-treatment scenario, cells were exposed to the salts for 24 hours before infection. In the therapeutic (post-treatment) approach, cells were first infected with CHIKV for an hour and then subjected to 24 hours of drug treatment. In the co-treatment approach, the virus was incubated with salts for an hour, and the cells were infected with the virus-drug mixture. After one hour, the mixture was removed, the cells were washed with sterile PBS, and incubated for 24 hours in MEM with 2% FBS. For all conditions, virus control (VC) wells were maintained in which the infected cells did not receive any treatment. After incubation, virus quantification was performed by assessing the viral load in the culture medium using the Foci Forming Unit (FFU) assay as per previously reported protocols and conditions (Alagarasu et al., 2022; Kasabe et al., 2023).

### 2.5 Dose dependent anti-CHIKV activity of zinc and magnesium salts

The dose-dependent effects of zinc and magnesium salts were evaluated under post-treatment conditions. Vero cells were infected with a 0.01 MOI of CHIKV at 37°C for 1 hour. After infection, cells were washed twice with 1x sterile PBS, followed by treatment with different concentrations of salts (ranging from 10 to 100 μM) dissolved in MEM with 2% FBS. The plates were then incubated for 24 hours, with a virus control maintained without salts. After incubation, the plates were freeze-thawed, and the clarified culture supernatant was assessed for infectious virus titre using the FFU assay and for viral RNA using the RT-qPCR method as per previously reported protocols and conditions (Alagarasu et al., 2022; Kasabe et al., 2023).

The percentage reduction in FFU titre compared to the virus control was calculated for each concentration, and the 50% inhibitory concentration (IC50) was determined using non-linear regression analysis with GraphPad Prism software version 9.

### 2.6 Effect of zinc and magnesium salts on the percent infectivity in Vero CCL81 cells

To assess the effect of zinc and magnesium salts on CHIKV infectivity, an immunofluorescence assay (IFA) was conducted. Vero cells were seeded onto sterile cell culture coverslips in a 24-well plate at a density of 2 × 10^5^ cells per well. The confluent monolayer of Vero cells was infected with a 0.01 MOI of CHIKV at 37°C for 1 hour. Following infection, cells were washed twice with 1x sterile PBS, after which different concentrations of salts dissolved in MEM with 2% FBS were added, while virus control was maintained without salts. The infected cells were visualized by staining with an in-house prepared anti-CHIKV monoclonal antibody (ClVE4/D9 clone) following incubation with a secondary antibody (anti-mouse IgG conjugated with FITC). The detailed assay was performed according to previously reported protocols and conditions (Alagarasu et al., 2022; Kasabe et al., 2023). Infected cells were counted, and percent infected cells were calculated for each concentration of salts with reference to the VC.

### 2.7 Effect of zinc and magnesium salts on the percent cell viability in CHIKV infected cells

The antiviral activity of the salts was further confirmed by investigating whether treatment with zinc and magnesium salts was able to prevent virus-induced cytopathic effects using the MTT assay. Infected cells treated with and without zinc and magnesium salts (2.5, 5, 10, 20, 40, 60, 80, and 100 μM concentrations) were subjected to the MTT assay, and percent cell viability was calculated with reference to uninfected and untreated cells.

### 2.8 Viral attachment and entry assay

Virus attachment and entry assays were performed to investigate the effect of zinc and magnesium salts on viral attachment and entry. In the attachment assay, salts were mixed with 1 MOI of CHIKV and incubated at 37°C for 1 hour, and Vero CCL-81 cells were infected with this virus-salt mix following 1 hour of incubation at 4°C. After 1 hour, cells were washed twice with 1x PBS and then incubated in 300 µl of MEM supplemented with 2% FBS at 37°C for 8 hours.

In the entry assay, Vero CCL-81 cells were first infected with CHIKV and incubated at 4°C for 1 hour, then washed twice with 1x sterile PBS. Zinc and magnesium salts were added to the cells, followed by incubation at 37°C with 5% CO2 for 2 hours. Afterward, cells were washed twice with 1x sterile PBS and incubated in 300 µl MEM supplemented with 2% FBS at 37°C for 8 hours.

A virus control without any salt was maintained for both the attachment and entry assays. Following incubation, the freeze-thawed clarified culture filtrates were used to determine the virus titre using an FFU assay. The FFU titres between salt-treated infected cultures and the virus control were compared to assess the effect of zinc and magnesium salts on virus attachment and entry.

### 2.9 Effect of time of exposure to zinc and magnesium salts and time of treatment initiation on CHIKV infection and replication

To ascertain the duration of exposure at which the salts are effective in inhibiting CHIKV replication, time of addition (TOA) and time of removal (TOR) assays were performed. Vero CCL-81 cells were cultured in 24-well plates and incubated at 37°C under 5% CO2 to facilitate adherence and growth. Cells were exposed to a 1 MOI of CHIKV for one hour at 4°C to synchronize infection and one hour at 37°C. The highest non-toxic concentrations of zinc and magnesium salts were added at 1, 2, 3, 4, and 6 hours for TOA. For the TOR assay, the highest non-toxic concentrations of zinc and magnesium salts were added immediately after virus entry and removed at 1, 2, 3, 4, and 6 hours. The cultures were then incubated in 300 µl MEM supplemented with 2% FBS at 37°C for an additional 8 hours in both cases. Positive controls with only virus-infected cells (no salts) and negative controls with uninfected cells were included for each time point with respect to TOA or TOR. After incubation, cells were freeze-thawed, and clarified culture supernatant was assessed for virus titre using an FFU assay.

### 2.10 Viral entry bypass assay

To find out whether the salts affect post-entry stages of the virus life cycle, an entry bypass assay was conducted in which the infection step was replaced by transfection of the cells with viral RNA. Vero CCL-81 cells (2×10^5^ cells per well) were added in 24-well plates and incubated overnight in 2% FBS MEM medium at 37^°^C. CHIKV RNA was extracted using the RNeasy kit as per manufacturer guidelines (QIAGEN), and the transfection complex was prepared in a sterile microcentrifuge tube by combining 40 μl (≥ 2 MOI of virus titre equals to 23 ng of viral RNA) of CHIKV RNA solution with 10 μl of Lipofectamine^®^ 2000 (Catalog No. 11668-019). The solution was mixed gently by pipetting up and down and incubated for 10 minutes at room temperature to allow the lipofectamine to complex with RNA. Opti-MEM media (50 μl) was added to the RNA tube and incubated for 30 min to form the lipofectamine-RNA complex. The growth media from the cells was carefully removed, followed by the dropwise addition of 100 μl of transfection complex to the cells and incubation at 37^°^C for one hour. Transfection media was removed, and salts were added and incubated for 24 hours. The freeze-thawed and clarified culture supernatants were analyzed for the expression of the CHIKV genomic RNA and negative strand RNA, which is the replication intermediate (Kaur et al., 2013). In the negative strand PCR, only a forward primer was used for reverse transcription, and for subsequent real-time PCR, both primers were used as described earlier. A FFU assay was performed to measure the titre of virus particles.

### 2.11 Effect of metallic salts on viral replication in mice

A total of 12 female C57BL/6 mice aged 3-4 weeks, obtained from our institutional animal breeding facility, were used in the study. The animals were categorized into four groups: virus control, ZnSO_4_, ZnAc, and MgSO_4_ treatment groups, each comprising three individuals (n = 3). The experiment was conducted twice for consistency in the results. These mice were chosen randomly, marked for individual identification, and housed in their cages for a minimum of one week before dosing. This acclimatization period allowed them to adapt to the laboratory conditions. To evaluate the potential anti-CHIKV effects of zinc and magnesium salts, the mice were infected with 1 × 10^7^ FFU/ml of CHIKV through the intramuscular route. Zinc and magnesium salts were dissolved in the Milli-Q water and given orally. A dose of 30 mg/kg was given on the alternative days for 7 days, as described earlier (Davis et al., 2022). Subsequently, viral RNA copies were quantified in serum on the second, third, fifth, and seventh day post-infection (dpi). The animals were humanely euthanized, followed by cervical dislocation at the end of the study.

### 2.12 Molecular docking of metallic salts to CHIKV proteins

Molecular docking studies were performed on the metal ion-binding server (MIB2) to find out the interactions of zinc and magnesium ions with CHIKV proteins (http://bioinfo.cmu.edu.tw/MIB2/) (Lin et al., 2016; Lu et al., 2022). Crystal structures of mature CHIKV envelope protein (3N42) (Voss et al., 2010), nsp2 protease (4ZTB) (Narwal et al., 2018), nsp2 helicase (6JIM) (Law et al., 2019), nsp4 RdRp domain (7F0S) (Tan et al., 2022), and nsp3 ADP ribose macrodomain (3GPO) (Malet et al., 2009) were obtained from the RCSB protein data bank in PDB format and uploaded to the MIB2 online server. The proteins showing the highest binding scores with the Zn and Mg salts were analyzed for interacting residues in the active site, and their interactions with the ions were visualized using Biovia Discovery Studio version 21.1.

### 2.13 Statistical analysis

Statistical analysis was performed using GraphPad Prism 9. Data were pooled from three independent experiments in triplicates (n=3/treatment groups per experiment) for analysis and reported as the mean ± SE. A non-linear regression analysis was performed to calculate the CC50 and IC50 values. The mean virus titre was represented as FFU/ml, RNA copy number, and percent infected cells and was compared using a one-way ANOVA. The results were considered statistically significant when the P values were less than 0.05.

## 3 Results

### 3.1 Cytotoxic effects of zinc and magnesium salts in Vero CCL-81 cells

The cytotoxicity of zinc and magnesium salts to Vero CCL-81 at concentrations ranging from 2.5 to 400 µM in Vero-CCL81 cells was assessed at 24-, and 48-hour time points using the MTT assay. Zinc sulphate and zinc acetate at concentrations up to 100 µM showed no toxicity, while concentrations higher than 100 µM exerted more than 20% cytotoxicity. Magnesium sulphate exhibited no observable toxicity at all the time points studied. The CC50 values in µM corresponding to various salts are shown in **Supplementary Figure 1**. The concentration of the salts that facilitated cell viability of ≥80% was subsequently selected for investigating their antiviral properties.

### 3.2 Zinc and magnesium salts exert anti-CHIKV activity under post-treatment conditions

**Figure 1:**
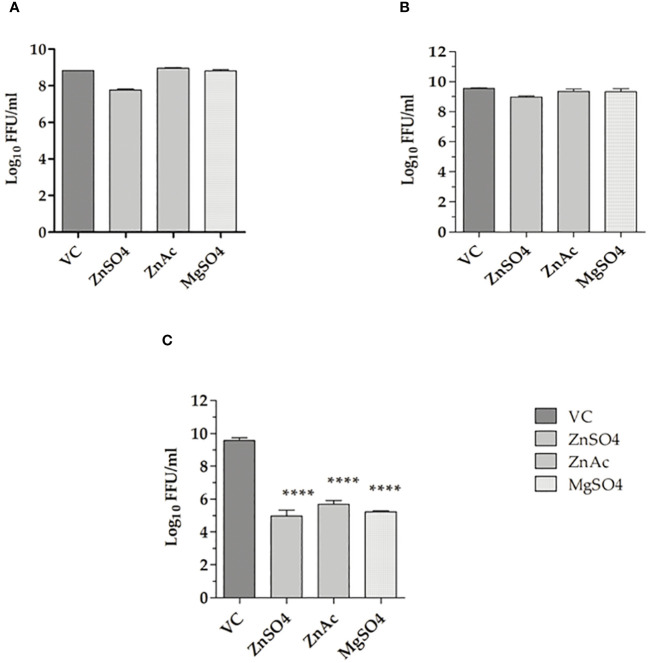
Effect of metallic salts on CHIKV replication under various treatment approaches. **(A)** Pre-treatment, **(B)** Co-treatment, and **(C)** Post-treatment. The experiments were performed in triplicates in two independent trials, and the virus titres are expressed as mean log_10_ FFU/ml ± SE. The virus output in cultures treated with zinc and magnesium salts were compared with the virus control (VC). ****p < 0.0001.

The prophylactic (pre-treatment), virucidal (co-treatment), and therapeutic (post-treatment) activities of the salts were assessed at concentrations of 100 µM. The metallic salts had no effect on virus titre under pre-treatment and co-treatment conditions (**Figures 1A, B**). Under post-treatment conditions, a notable reduction (≥1 log10 reduction) in CHIKV titre was noticed in cultures that received the salts compared to the virus control (infected cells without any treatment). In cultures treated with zinc sulphate a reduction in virus titre from 9 ± 0.5 to 5 ± 0.5 mean log_10_ FFU/ml was observed. In cultures treated with zinc acetate, the virus titre was reduced to 4 ± 0.5 mean log_10_FFU/ml in treated cultures from 9 ± 0.5 mean log_10_FFU/ml in VC. In cultures that received magnesium sulphate, a four-log decrease in titre was observed (9 ± 0.5 to 5 ± 0.5 mean Log_10_FFU/ml) (**Figure 1C**).

### 3.3 Anti-CHIKV activity of zinc and magnesium salts in a dose-dependent manner

**Figure 2:**
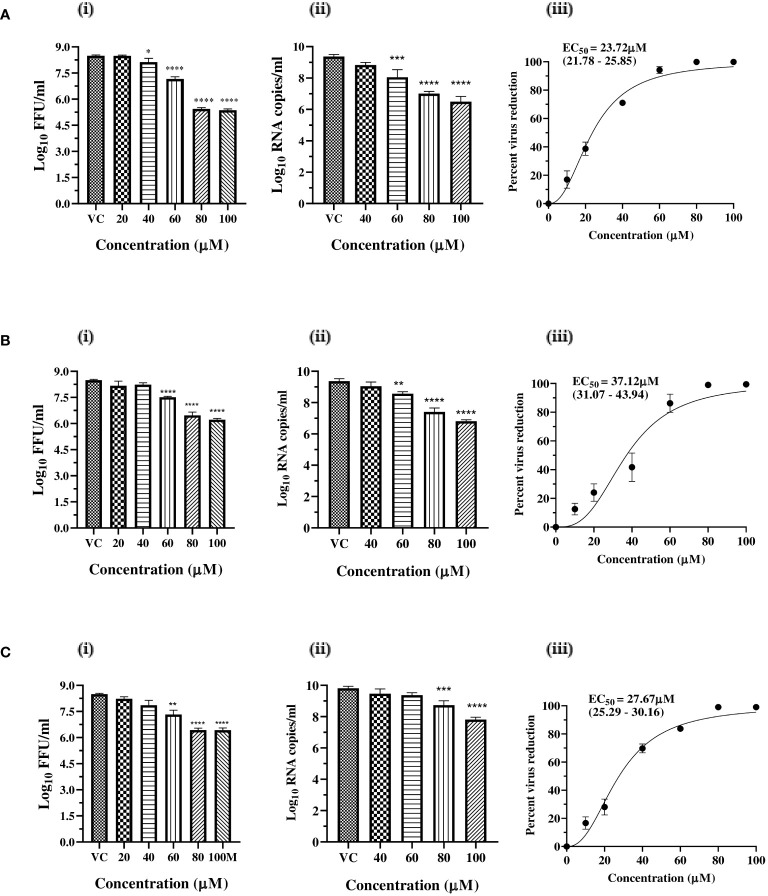
Dose dependent antiviral effects of **(A)** zinc sulphate, **(B)** zinc acetate, and **(C)** magnesium sulphate on CHIKV infection and replication. (i) Effect of treatment of Vero CCL-81 cells with zinc and magnesium salts on the production of infectious virus titre expressed as mean log_10_ FFU/ml ± SE (ii) Effect of treatment of Vero CCL-81 cells with zinc and magnesium salts on the synthesis of genomic RNA expressed as mean log_10_ viral RNA copy number/ml ± SE. (iii) Mean percent virus reduction ± SE based on FFU titre exerted by different concentrations and IC50 (95% CI). ** p<0.01; *** p<0.001; **** p<0.0001.

Since a reduction in virus titre was observed under post-treatment conditions, the dose-dependent antiviral activity of zinc and magnesium salts was investigated, and the virus titre was assessed. The results revealed a concentration-dependent decrease in virus titre in terms of FFU/ml, RNA copy number/ml, and percentage of infected cells, in cultures treated with the salts compared to the VC. Based on FFU titre, the percentage virus reduction, and the IC50 values, which represent the concentration required to inhibit 50% of the virus titre, were calculated. The IC50 values for zinc sulphate, zinc acetate, and magnesium sulphate were 23.72 µM, 37.12 µM, and 27.67 µM, with selectivity index (SI) of 11.10, 6.7, and 76.72, respectively (**Figures 2A–C**).

### 3.4 Zinc and magnesium salts reduce virus infectivity

**Figure 3:**
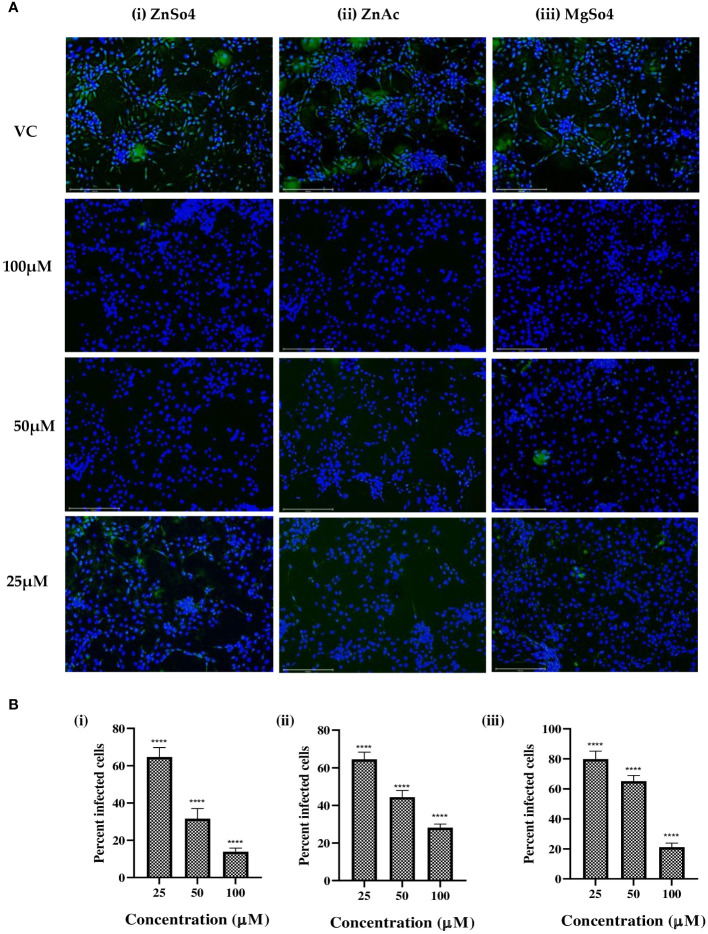
**(A)** Immunofluorescence images of CHIKV-infected Vero cells treated with different concentrations of (i) zinc sulphate, (ii) zinc acetate, and (iii) magnesium sulphate. Infected cells appear as green due to expression of capsid protein which is probed with a FITC labelled monoclonal antibody against the capsid protein, while uninfected cells appear as blue due to nuclear staining with DAPI. **(B)** Effect of (i) zinc sulphate, (ii) zinc acetate, and (iii) magnesium sulphate treatment on percent cell infection in Vero CCL-81 cells. **** p<0.0001.

To validate the antiviral effect of zinc and magnesium salts, IFA was performed to quantify the percent infection and the capsid protein levels of CHIKV in infected and treated cells. A dose-dependent decrease in viral protein expression was observed in infected cultures that received zinc and magnesium treatment (**Figures 3A, B**).

### 3.5 Zinc and magnesium salts rescue cell viability in CHIKV infected cells

To find out whether treatment with metallic salts protects against virus-induced cytopathic effects, the cell viability in infected cell cultures treated with and without zinc sulphate, zinc acetate, and magnesium sulphate relative to the uninfected and untreated cells was assessed. It was observed that higher non-toxic concentrations of all the metallic salts rescued 100% cell viability in infected cell cultures compared to VC (**Supplementary Figures S2**).

### 3.6 Zinc salt inhibits CHIKV entry

**Figure 4:**
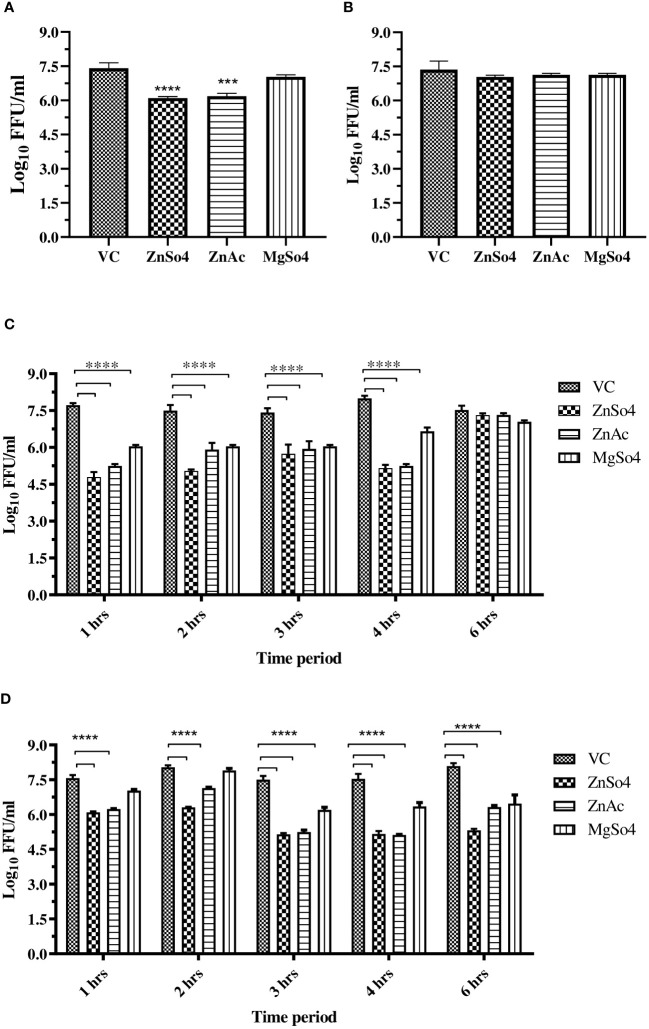
**(A)** Entry assay, and **(B)** Attachment assay, **(C)** Effect of time of addition of zinc and magnesium salts to CHIKV infected Vero CCL-81 cells on virus titre. **(D)** Effect of exposure time (time of removal) of zinc and magnesium salts to CHIKV infected Vero CCL-81 cells on virus titre. The experiments were conducted in triplicate and independently repeated twice. The virus titre was represented as mean log_10_ FFU/ml ± SE. All treatment groups were compared against the control group (VC) consisting of infected cells without treatment. **** p<0.0001.

To investigate the potential effects of zinc and magnesium salts during virus attachment, entry, and uncoating, entry and attachment assays were conducted. Virus attachment was facilitated by incubating cells with virus and salt mixtures at 4°C following a one-hour incubation at 37°C. A significant reduction (≥1 log10 reduction) in CHIKV titre was observed in cultures treated with the salts compared to the virus control, suggesting that zinc salts inhibited virus entry. However, both the salts were not effective during virus attachment (**Figures 4A, B**).

### 3.7 Zinc and magnesium salts inhibit CHIKV replication

TOA and TOR assays were conducted to investigate the impact of exposure duration and timing on the inhibitory effects of zinc and magnesium salts on CHIKV infection and replication in Vero CCL-81 cells. The experiments revealed that zinc treatment within 4 hours after virus entry, with a minimum exposure of 1 hour, significantly reduced virus titers compared to the control (VC). whereas magnesium salt treatment required a minimum exposure of 3 hours and administration within 4 hours after virus entry to effectively inhibit CHIKV in cultures treated with magnesium salts compared to the control (VC) (**Figures 4C, D**).

### 3.8 Zinc and magnesium salts inhibit CHIKV during post-entry stages

**Figure 5:**
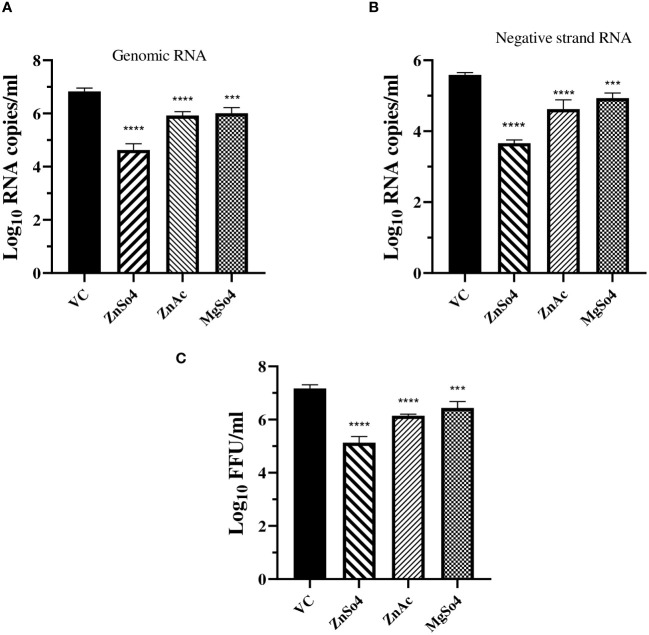
Effect of zinc and magnesium salts on the production of genomic RNA **(A)**, negative strand RNA **(B)**, and infectious virus particles **(C)**, when the cells were transfected with viral RNA. The virus output was expressed as mean log _10_ RNA copy number or FFU/ml ± SE One-way ANOVA test was used to compare the differences between treated and untreated cultures with statistical significance indicated as *** p<0.001; **** p<0.0001.

To find out whether the zinc and magnesium salts act at the level of entry and uncoating or at post-entry stages, a entry bypass assay was performed. The culture filtrates were investigated for the presence of genomic RNA, negative-strand RNA, which is a marker of viral replication, and infectious virus titre. A significant decrease in genomic RNA was observed in the cultures treated with zinc and magnesium salts (**Figure 5A**). Compared to transfected cultures that did not receive any treatment, a lower copy number of CHIKV negative strand RNA was observed in cultures treated with all the salts studied. However, the effect was more pronounced in cultures treated with zinc sulphate, followed by zinc acetate and magnesium sulphate (**Figure 5B**). A significant decrease in the infectious virus titre was noticed in cultures treated with zinc and magnesium salts, and the effect was more pronounced in cultures that received zinc sulphate treatment (**Figure 5C**). The results suggest that zinc salts affect the post-entry stages.

### 3.9 Treatment of infected mice with zinc and magnesium salts decreased viral load in serum

**Figure 6:**
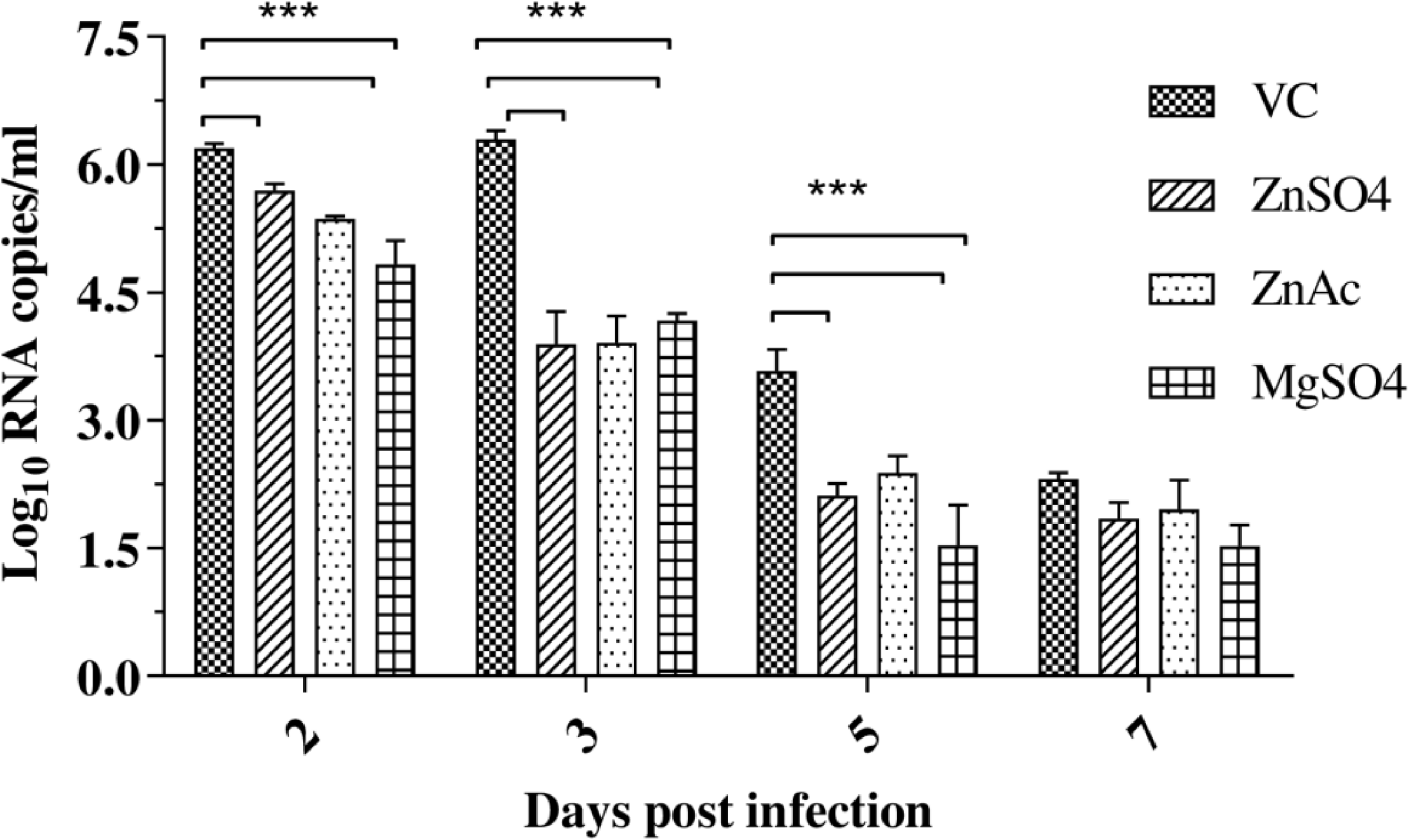
Effect of zinc sulphate, zinc acetate and magnesium sulphate (30mg/kg) administered orally to C57/BL6 mice on serum viral RNA load. The viral RNA copies were measured on the 2^nd^, 3^rd^, 5^th,^ and 7^th^ day after infection. The results are presented as log_10_ RNA copies/ml ± SE. Statistical significance was denoted as ***p<0.001.

Oral feeding (30 mg/kg) of CHIKV-infected mice with zinc sulphate, zinc acetate, and magnesium sulphate significantly reduced the viral RNA load in serum. At 2^nd^ dpi, magnesium sulphate-treated groups showed one log_10_ reduction (99% reduction); at 3^rd^ dpi, all the treatment groups showed 2.1 log_10_ reduction (99.2% reduction) of viral RNA compared to animals (VC), which did not receive the salts (*p* < 0.05). At 5^th^ dpi, the reduction in viral RNA copy number was 1.8 log_10_ (*p* < 0.05). At 7^th^ dpi, no difference in the viral RNA load was observed between treated and untreated animals. The therapeutic effect was more pronounced for magnesium sulphate on the 2^nd^ day, while the effects of both zinc and magnesium salts were the same from the 3^rd^ day onwards (**Figure 6**).

### 3.10 Molecular interaction of zinc and magnesium ions with CHIKV proteins

**Figure 7:**
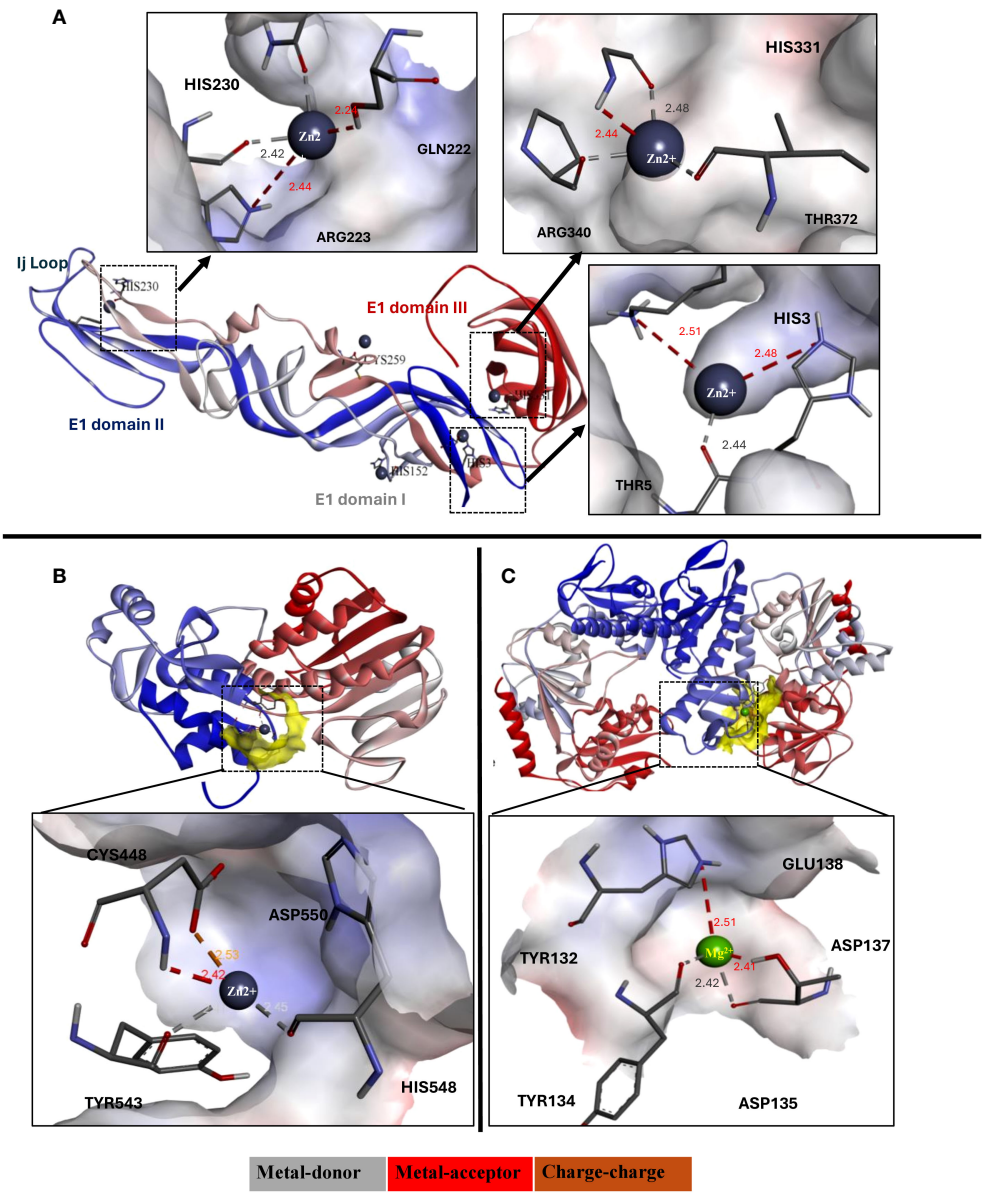
**(A)** Ribbon diagram (3d view) of E1 domain of CHIKV envelope glycoprotein interacting with zinc ions, and their detailed interaction and binding properties. **(B)** Ribbon diagram (3d view) of CHIKV protease interacting with zinc ions, and **(C)** Ribbon diagram (3d view) of CHIKV helicase interacting with magnesium ions. The ribbon structure of proteins depicted in blue to red color represents N-terminus to C-terminus sequence.

Molecular docking of CHIKV proteins with zinc and magnesium ions was performed using the MIB2 server. Docking results of zinc ions with the envelope proteins suggested strong interaction with the conserved residues of the E1 domain including His3, Asp75, Asn100, His152, His230, and His331 (**Figure 7A**). Moreover, the results showed that zinc ions bound best to the CHIKV nsP2 protease and magnesium ions bound best to the helicase domain compared to other structural and non-structural proteins. The highest possible interaction of Zn ions involved the active site amino acid residues Cys448, Glu520, His548, and Asp550 (**Figure 7B**), and the highest possible interaction of Mg ions involved the residues Tyr132, Asp135, Asp137, and Glu138 (**Figure 7C**). The detailed binding score and interacting residues are described in **Supplementary Table 1**.

## 4 Discussion

The present study was carried out to explore the anti-chikungunya activity of zinc and magnesium salts. The results revealed the potential antiviral effects of zinc sulphate, zinc acetate, and magnesium sulphate against CHIKV *in vitro* and *in vivo*. The findings were confirmed by evaluating the titre of infectious virus particles using the FFU assay, the genomic viral RNA copy number and negative strand RNA copy number using qRT-PCR, and the percent of cells infected by immunofluorescence assay.

Earlier studies have shown that chelation of zinc, leading to its deficiency, induces oxidative stress, which specifically affects positive-strand RNA viruses, including CHIKV (Khan et al., 2021). Zinc ions have been reported to inhibit the pH-dependent liposomal fusion of the E1 domain of alphavirus envelope glycoproteins by binding with high affinity to histidine, cysteine, and asparagine residues (Bracha and Schlesinger, 1976). The entry assay revealed that zinc might interfere with virus entry and uncoating, a finding further supported by molecular interaction studies of zinc ions with E1 and E2 glycoproteins. Zinc ions exhibited high affinity for His3, His230, and His331, which are conserved in the alphavirus E1 domain and play a critical role in the fusion process. This affinity might have inhibited the fusion-infection process by competing for protonation of these histidine residues, similar to what was described for Semiliki forest virus (Corver et al., 1997).

In the present study, supplementation with zinc and magnesium salts affected CHIKV post-entry stages of virus life cycle. Zinc acetate inhibits the nSP2 protease activity of CHIKV (Saha et al., 2018), which was confirmed in the *in silico* experiments of the present study. The zinc ion forms a rigid coordination with Cys448, Asp538 and His548 with a bond length of <2.5A°, suggesting possible inhibition of the active site of the CHIKV protease. Similar results have been observed in many studies, suggesting zinc as a potent inhibitor of the nSP2 protease (Saisawang et al., 2015). Studies have reported that one cycle of CHIKV replication and virion formation takes 5–6 h post infection (Reis et al., 2022). TOA, TOR, and entry bypass studies showed that zinc and magnesium salts might have inhibited CHIKV post-entry stages, including proteolytic processing of polyproteins, replication, and assembly of virions, which required treatment initiation before 4 hours of post-infection. Though zinc salts reduced CHIKV titre even within one hour of exposure, the maximum reduction in virus titre was observed in cultures exposed for atleast three hours after infection. This observation suggests that zinc salts might act at multiple steps after infection including entry, proteolytic processing of polyproteins, replication, and assembly of virions. The inhibitory effect of zinc salts was more evident against the synthesis of negative strand RNA, indicating that zinc salts might affect replication or pre-replication stages such as proteolytic processing. Treatment after 4 hours of virus entry had no effect on virus titre, suggesting that zinc and magnesium salts may not affect the viral assembly and release. Among the zinc salts, zinc sulphate was more effective in inhibiting CHIKV compared to zinc acetate. Zinc sulphate (23% zinc) is generally more soluble, and bioavailability is high compared with other forms of zinc (Herman et al., 2002). The bioavailability of zinc acetate and zinc sulphate might be the same, but there is no human data available (Wegmüller et al., 2014). Even though both compounds release zinc ions, the rate at which these ions are released and their availability for cellular uptake can vary.

The finding that magnesium sulphate inhibits CHIKV is a serendipitous discovery, and the mechanism of antiviral activity is not clearly understood. The antiviral activity of magnesium sulphate against mouse hepatitis virus has been reported (Mizutani et al., 1994). *In silico*, docking of magnesium ions with CHIKV proteins revealed that it binds to the nSP2 helicase. However, it has been shown that Mg2+ ions are required for nSP1 methyl transferase and guanylylation activities and nSP2 helicase nucleoside triphosphatase function (Karpe et al., 2011; Kaur et al., 2018). At the same time, magnesium has been reported to have a role in ~600 enzymatic reactions (de Baaij et al., 2015). This suggests that magnesium sulphate might exert antiviral activity through host-dependent mechanisms rather than interfering with the activity of viral proteins. However, the findings from the different time dependent assays and the entry bypass assay suggest that magnesium salt effects only post-entry stages or during replication of CHIKV. Alternatively, high concentrations of magnesium might affect viral protein activity through feedback regulation. The antiviral activity post-infection also indicates that host mechanisms that function to suppress viral replication might be potentiated by magnesium ions. Magnesium sulphate might exert a distinct mechanism that may not involve direct interference with viral entry, and this needs further investigation.

*In-vivo* studies using C57BL6 mice revealed that oral feeding of infected mice with zinc and magnesium salts reduced the initial viral RNA load, though, at 7^th^ dpi, the viral RNA copy numbers (~100 copies) were similar in treated and untreated mice. However, in treated mice, the reduction in viral RNA copy number was comparatively faster and reached ~100 copies in 5^th^ dpi, while it occurred at 7^th^ dpi in untreated mice, suggesting that feeding with zinc and magnesium salts might lead to rapid clearance of viruses, thereby reducing inflammation and subsequent sequelae. The main limitation of the *in-vivo* study is that the mouse model used in the study did not develop any clinical symptoms to investigate the effect of salts on the clinical course of the disease, including arthritis. Further the studies on inflammatory response in the infected mice need to be conducted.

Plasma zinc levels range between 9 and 20 µmol/L. The human zinc half-life is approximately 280 days (Nriagu, 2011). According to the Toxnet database of the U.S. National Library of Medicine, the oral LD50 for zinc, measured in rats and mice, is close to 3 g/kg body weight (Coni et al., 2021). In the present study, zinc concentrations ranging from ~17 to 45 µmol are required for a 50% reduction in the virus titre *in-vitro* when added at different time points. This concentration mimics the normal plasma concentration. The safety profile of high-dose intravenous zinc (HDIVZn) has been well documented in the literature. In the treatment of burns, HDIVZn has been administered at doses ranging from 26.4 to 37.5 mg/day for eight consecutive days without any reported side effects (Berger et al., 2007; Cvijanovich et al., 2016). Notably, oral zinc doses exceeding 75 mg/day have demonstrated antiviral effects against common cold viruses, including influenza viruses (Hemilä, 2017). Mild adverse effects associated with zinc supplementation have been reported at dosages surpassing 200 mg/day (Fosmire, 1990).

## 5 Conclusion

Since viral persistence is one of the contributors to chikungunya-associated chronic arthritis, early treatment with zinc and magnesium salts might help in the clearance of the virus and prevent the subsequent development of arthritis. However, further trials in humans are needed. To conclude, the current study reports that zinc and magnesium salts exhibit anti-CHIKV activity, which might have implications for designing therapeutic strategies against CHIKV infection and the development of CHIKV-associated arthritis.

## Data availability statement

The original contributions presented in the study are included in the article/**Supplementary Material**. Further inquiries can be directed to the corresponding authors.

## Ethics statement

The animal study was approved by Committee for Control and Supervision of Experiments on Animals (CCSEA) (IAEC number CHK 1501, approved on December 20, 2017). The study was conducted in accordance with the local legislation and institutional requirements.

## Author contributions

KD: Formal analysis, Investigation, Visualization, Writing – original draft, Data curation, Methodology, Software, Validation. SS: Data curation, Formal analysis, Investigation, Methodology, Software, Validation, Writing – original draft. MK: Formal analysis, Methodology, Writing – review & editing. SC: Writing – review & editing, Investigation, Project administration, Resources, Software, Supervision. KA: Investigation, Project administration, Software, Supervision, Writing – review & editing, Conceptualization, Data curation, Formal analysis, Visualization, Writing – original draft. DP: Conceptualization, Formal analysis, Investigation, Project administration, Supervision, Visualization, Writing – original draft, Writing – review & editing, Funding acquisition, Resources.
