# Supplementary material for: Explorations on the antiviral potential of zinc and magnesium salts against chikungunya virus: implications for therapeutics
Source: Front Cell Infect Microbiol. 2024 Jun 4;14:1335189. doi: 10.3389/fcimb.2024.1335189 (PMC11183322; doi:10.3389/fcimb.2024.1335189)
Supplement: Supplementary file 1 [file DataSheet_1.pdf]

Supplementary Material

Explorations on the antiviral potential of Zinc and Magnesium salts against chikungunya virus: Implications for therapeutics

Kusuma Sai Davuluri[1#], Shridhar Shukla[1#], Mahadeo Kakade[1], Sarah Cherian[2], Kalichamy Alagarasu[1]*, Deepti Parashar[1]*

Supplementary figures

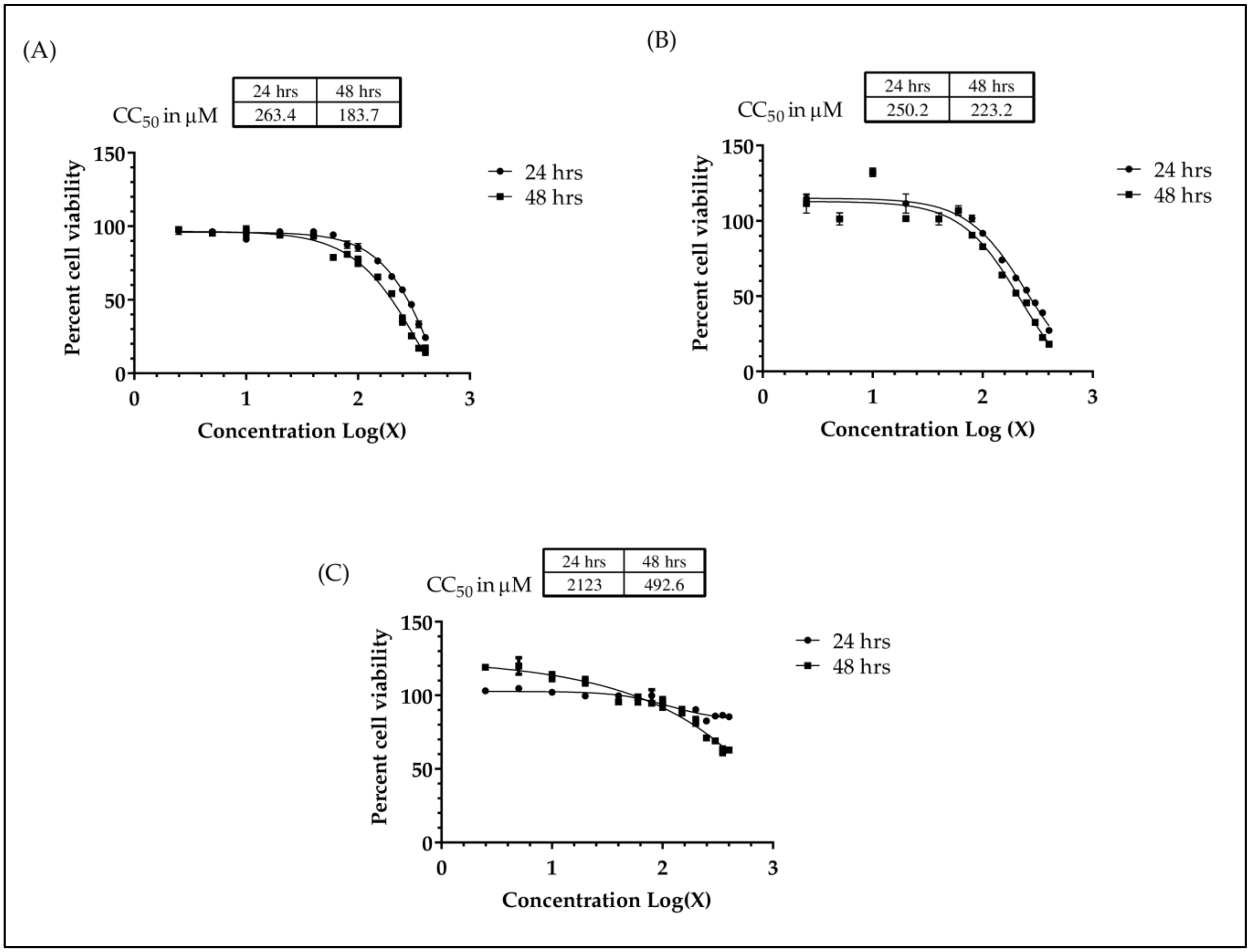

Supplementary figure 1: Effect of zinc and magnesium salts on the cell viability at different time points of incubation and CC50 Values. (A) Zinc sulphate, (B) Zinc acetate and (C) Magnesium sulphate. Percent cell viability was calculated with reference to cultures that did not receive any treatment.

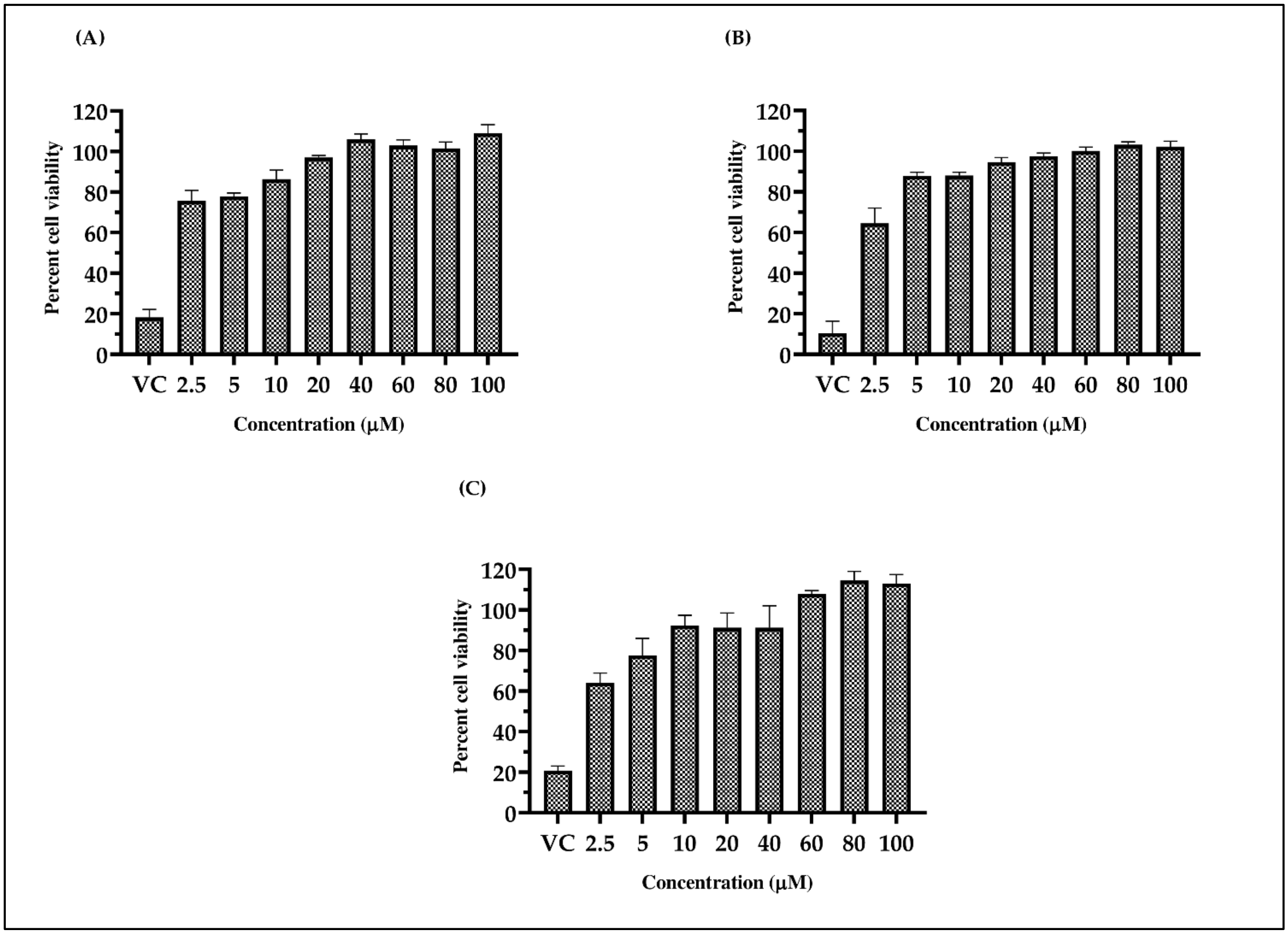

Supplementary figure 2: Effect of zinc sulphate (A), zinc acetate (B), and magnesium sulphate (C) on percent cell viability in CHIKV infected cells compared to VC. Percent cell viability in CHIKV infected cell cultures without any salts was used as reference.

Supplementary Table 1: Highest binding score of metal ions with conserved catalytic residues of respective CHIK proteins

**Sr. No**	**Metal ions**	**Envelope protein (3N42)**	**NSP3 ADP-ribose macrodomain (3GPO)**	**NSP4 RdRp domain (7F0S)**	**NSP2 protease (4ZTB)**	**NSP2 helicase (6JIM)**	**Interacting residues of CHIKV proteins with high binding score**
1	Zn^{2+}	3.4	2.9	3.2	4.31	2.8	**E1 glycoprotein-** HIS3, ASP75, ASN100, HIS107, ASP151, HIS152, HIS230, CYS301, HIS308, HIS331,<br><br>**NSP2 protease-** CYS448, GLU520, ASP531, TYR543, HIS548, ASP550, HIS610, HIS616, HIS 687,
2	Mg^{2+}	1.7	NA	2.3	2.9	6.505	**NSP2 helicase** TYR132, ASP135, ASP137, GLU138, VAL248, ARG288, PHE287, SER236,
